# Postnatal development of bones and related hormonal factors in piglets

**DOI:** 10.1093/jas/skag331

**Published:** 2026-10-10

**Authors:** Julia C Vötterl, Frederike Lerch, Fitra Yosi, Simone Koger, Barbara U Metzler-Zebeli

**Affiliations:** Centre for Animal Nutrition and Welfare, Clinical Department for Farm Animals and Food System Transformation, University of Veterinary Medicine, Vienna 1210, Austria; Centre for Animal Nutrition and Welfare, Clinical Department for Farm Animals and Food System Transformation, University of Veterinary Medicine, Vienna 1210, Austria; Centre for Animal Nutrition and Welfare, Clinical Department for Farm Animals and Food System Transformation, University of Veterinary Medicine, Vienna 1210, Austria; Department of Animal Science, Faculty of Agriculture, University of Sriwijaya, Palembang 30662, Indonesia; Centre for Animal Nutrition and Welfare, Clinical Department for Farm Animals and Food System Transformation, University of Veterinary Medicine, Vienna 1210, Austria; Centre for Animal Nutrition and Welfare, Clinical Department for Farm Animals and Food System Transformation, University of Veterinary Medicine, Vienna 1210, Austria

**Keywords:** bone growth, gene expression, histology, piglet, serum parameters

## Abstract

Piglets grow considerably in their first weeks of life. However, little information exists about the interplay of hormonal factors that orchestrate bone development. The present study aimed to investigate the development of hormonal factors in serum, bone metrics, histology and mRNA expression of genes related to growth and transformation of bone tissue from day of life (DoL) 3 to 35. Litters (*n *= 10) remained with their mother sows until weaning on DoL 28. Blood and metacarpal bones of 10 piglets (5 males and 5 females) were collected on DoL 3, 7, 14, 21, 28, 31, and 35. Bone geometry parameters linearly increased during the suckling phase (*P *< 0.05). Histological parameters, including the total width of the growth plate and its proliferative and reserve zones, in turn, quadratically changed from DoL 3 to 28 (*P *< 0.05), indicating greater longitudinal bone growth between DoL 7 to 14. Osteocyte and osteoclast numbers also linearly increased from DoL 3 to 28 along with increasing mRNA expression of *RANKL* and *OPG* (*P *< 0.05), indicating bone maturation. Serum alkaline phosphatase and bone-specific alkaline phosphatase, important factors for bone mineralization, were higher in the first week of life and linearly declined thereafter (*P *< 0.05). Serum levels of parathyroid hormone (PTH) and vitamin D, which respond to decreasing Ca levels in the blood, linearly increased during the suckling phase (*P *< 0.05). Serum levels and mRNA expression of fibroblast growth factor-23 (FGF23), that regulates the renal phosphate excretion, and of *KLOTHO*, needed for the action of FGF23 on the kidney, linearly decreased during the suckling phase (*P *< 0.05), which allowed the retention of phosphate for bone growth. Postweaning, bone geometry parameters suggested growth stagnation from DoL 28 to 35. However, quadratic effects of age on histological parameters for the growth plate indicated interrupted growth mainly on DoL 31, whereas on DoL 35 longitudinal bone growth resumed (*P *< 0.05). Quadratic effects of age on postweaning levels of serum Ca and phosphate and expression of *KLOTHO* supported reduced growth on DoL 31 (*P *< 0.05), whereas serum levels of vitamin D and parathormone linearly increased and decreased postweaning, respectively (*P *< 0.05). Overall, the present data provide valid information on bone mineralization and development of the regulatory interplay of hormonal factors and feedback loops during the suckling phase and the impact of weaning on bone growth.

## Teaser

The present results expand our understanding of bone development in newborn piglets. They provide an insight into the interplay of hormonal factors in serum and at the bone level that are associated with bone mineralization and remodeling processes in newborn piglets from d 3 of life until 1 wk after weaning.

## Introduction

Disturbances in bone and cartilage formation are not uncommon in pigs due to the high growth rate of modern pig breeds (Tomaszewska et al. 2013). Bones comprise 98% and 80% of the body’s total calcium (Ca) and phosphorus (P), respectively. Therefore, appropriate supply of Ca and P is mandatory to ensure sufficient mineralization of the rapidly growing bones (Wang et al. 2023). Despite the importance of proper development for lifelong skeletal health of pigs, reference values for bone density and mineralization mainly exist for nursery and growing pigs and sows (Sørensen et al. 2018; Gerlinger et al. 2021; Lee et al. 2022), whereas data are scarce for neonatal pigs (Witschi et al. 2011; Williams et al. 2023, 2024). In older animals, bone mineralization and bone density are determined using imaging technologies and/or chemical assessment of the bones (Williams et al. 2023, 2024). They require the availability of expensive technical equipment, transport and sedation or euthanasia of the lame animal and are also less applicable for monitoring bone development in suckling piglets on the farm.

Besides its static function, the bone is a metabolically active tissue and requires control through hormonal factors. Thus, measuring factors that regulate bone formation and remodeling processes in the blood may be an alternative approach to assess bone development in young piglets. There is currently little information on ranges of bone biomarkers in suckling piglets which are used in older pigs, such as osteocalcin (**OCN**) and the bone-specific alkaline phosphatase (**BALP**) (Sørensen et al. 2018; Lee et al. 2020; Jaramillo et al. 2025). Both factors are released from osteoblasts (Lee et al. 2020; Jaramillo et al. 2025). When carboxylated, OCN appears to have the ability to chelate and import Ca from the blood to the bone (Aaseth et al. 2024). Bone-specific alkaline phosphatase promotes bone mineralization by providing an alkaline environment for Ca crystallization and by blocking inhibitors (Basu et al. 2013; Bouksila et al. 2019; Kikyo 2024). It represents a proportion of the total detectable alkaline phosphatase (**ALP**) in the blood (Bartlett et al. 2022; Brown et al. 2022). Other central regulators of the Ca and P homeostasis are the hormones vitamin D (**VitD**) and parathormone (**PTH**), which are often also tested when assessing the mineral status in pigs (Williams et al. 2023, 2024). Another important factor for the Ca and P homeostasis is fibroblast growth factor 23 (**FGF23**), which is produced by osteocytes and osteoblasts (Courbebaisse and Lanske 2018) but less investigated in relation to bone development in pigs so far. In older animals, FGF23 secretion is stimulated by 1,25-hydroxy-VitD and high phosphate levels in the blood (Szeliga et al. 2024). Available data for suckling piglets show a decline of ALP, BALP, and OCN during the suckling phase, whereas PTH and VitD remained stable or slightly increased over time (Zhang et al. 2019; Metzler-Zebeli et al. 2023; Yosi et al. 2025). Postweaning, serum ALP and FGF23 continues to decline over time, whereas OCN and VitD showed quadratic age-related responses, which were influenced by the dietary P availability (Vötterl et al. 2021). Overall, little is known about the ranges and interplay of the above-mentioned factors in suckling piglets. This is important information not only for monitoring bone development but also for evaluating nutritional strategies to promote bone and joint health in young pigs. Therefore, the present study aimed to investigate the development of bones, hormonal factors in serum and expression of genes related to growth and transformation of bone tissue from day of life (**DoL**) 3 until the early postweaning phase on d 35 of life. We hypothesized that serum levels of hormones related to bone metabolism show clear age-dependent patterns during the suckling phase that correspond to the stage of bone maturation visible at the histological and gene expression level. Moreover, we assumed that serum parameters and bone metrics would clearly reflect growth stagnation during the immediate time after weaning.

## Material and methods

### Ethical statement

All procedures involving animal handling and treatment were approved by the institutional ethics committee of the University of Veterinary Medicine Vienna and the National authority according to the Law for Animal Experiments, Tierversuchsgesetz (GZ 2020-0.437.208).

### Animals, housing, and experimental procedures

The bone samples were collected in a previous experiment on gastrointestinal development of suckling and newly weaned piglets (Yosi et al. 2005, 2024). Accordingly, the experimental setup has been described in detail elsewhere (Yosi et al. 2024). Bone samples were collected from piglets (Large White × Piétrain) of 10 Large White sows in the second replicate batch of the aforementioned study. The experiment was conducted at the pig facility of the University of Veterinary Medicine Vienna (Vetfarm) from farrowing until 7 d after weaning. Weaning occurred on DoL 28. Housing and management of sows and piglets corresponded to the standard protocol at the pig facility. The sows were individually housed in free farrowing pens (Befree, Schauer, Agrotonic, Prambachkirchen, Austria; 2.3 m × 2.6 m) from 5 d before farrowing throughout the suckling period. Each farrowing pen was equipped with a trough, hay rack and a bowl drinker for the sows and a round feeder, small bowl drinker and a nest with heated flooring for the piglets. The piglets remained with their mother sow during the 4-wk lactation period and could suckle freely. At weaning, the piglets were moved to the nursery unit, where 20 piglets from 2 to 3 litters were housed together in one pen (3.3 m × 4.6 m). Each nursery pen was equipped with one round feeder, two bowl drinkers and a heated nest.

From DoL 3, piglets were offered a commercial milk replacer (Weanplus-4, Startix, Voorthuizen, The Netherlands; Table S1), which was freshly prepared at least twice daily according to the manufacturer’s instructions. The milk replacer was gradually replaced from DoL 24 by the prestarter diet (Königshofer GmbH, Ebergassing, Austria; Table S1). Blending started with a ratio of 70:30 (w/w) on DoL 24, 50:50 (w/w) on DoL 25, and 30:70 (w/w) on DoL 26. The prestarter diet was fed 100% as mash on DoL 27 and in dry form from DoL 28 to 35. The diets used in the present experiment met or exceeded the current recommendations for nutrient requirements (NRC 2012). Water was freely available to the sows and piglets.

### Blood and metacarpal bone sampling

Piglets were weighed at birth and on each of the 7 sampling days (DoL 3, 7, 14, 21, 28, 31, and 35). On each sampling day, blood and bones were collected from 10 piglets (5 males and 5 females); one piglet from each litter with average body weight (with alternating sexes on the consecutive sampling days). A blood sample was taken from the heart after deep sedation with azaperone (Stresnil 40 mg/mL, Elanco Tiergesundheit AG, Basel, Switzerland) and ketamine (Narketan 100 mg/mL, Vetoquinol Österreich GmbH, Vienna, Austria) and placed in serum tubes (Vacuette tube CAT Serum, Greiner Bio-One International GmbH, Kremsmünster, Austria). Blood samples were kept on ice until centrifugation at 3,000 × *g* for 20 min at 4°C (Eppendorf Centrifuge 5810 R, Eppendorf SE, Hamburg, Germany). The collected serum was stored at −80°C. After blood sampling, the animals were euthanized by intracardiac administration of embutramide (T61, Intervet GmbH, Vienna, Austria) and exsanguinated by cutting the major neck arteries and veins. The metacarpal bones of the right and left forelimbs were removed. For gene expression analysis, the metacarpal bones of the right side (third and fourth) were packed in sterile Whirl-Pak bags (Filtration Group GmbH, Öhringen, Germany), shock-frozen in liquid nitrogen and then stored at −80°C. The right (third) metacarpal bone of the left side was packed in Whirl-Pak bags and then stored at −20°C until metric bone parameters were determined. The left (fourth) metacarpal bone was placed in 50 mL tubes filled in neutral buffered 4% formalin solution and stored at 4°C until preparation of the histological sections for the examination of the bone structure.

### Serum analysis

The serum levels of phosphate, Ca and ALP were analyzed using enzymatic colorimetric assays on an autoanalyzer for clinical chemistry (Cobas 6000/c501; Roche Diagnostics GmbH, Basel, Switzerland). Porcine-specific enzyme-linked immunosorbent assays (**ELISA**) were used to determine FGF23 (Porcine FGF23 ELISA Kit, Fine Test, Wuhan Fine Biotech Co., Ltd, Hubei, China), BALP (Ostase BAP EIA [AC-20F1], Immunodiagnostic Systems Holdings PLC, Boldon, UK), OCN (N-MID Osteocalcin ELISA, Immunodiagnostic Systems Holdings PLC, Boldon, UK), 25-hydroxyvitamin vitamin D_3_ and D_2_ (25-hydroxyvitamin D EIA, Immunodiagnostic Systems Holdings PLC, Boldon, UK) and PTH (Intact PTH ELISA, Immunodiagnostic Systems Holdings PLC, Boldon, UK). The coefficients of variation for the various ELISA tests were <10%.

### Geometrical bone analysis

Geometry parameters were determined after thawing the fourth metacarpal bone. The length of the bone was determined using an electric caliper and the weight of the bone was determined using a precision scale (Mettler Toledo type MS205DU, Mettler-Toledo GmbH, Greifensee, Switzerland). The bone density was analyzed determining the water displacement using the Archimedes’ principle (bone density [g/cm^3^] = bone weight [g] × density of water [g/cm^3^]/water displacement [g]; Vötterl et al. 2020). The dry matter content of the bone was determined by drying the bone at 103°C for 24 h. After flaming the bones, they were incinerated in a muffle furnace (CWF 1100, Carbolite Gero GmbH & Co KG, Neuhausen, Germany) at 580°C for 24 h to determine crude ash.

### Histological bone analysis

The third metacarpal bone was decalcified for several weeks using ethylenediaminetetraacetic acid. After decalcification and dehydration, the bones were cut twice, vertically in the diaphysis of the bone and longitudinally across the growth plate. The individual pieces were dehydrated using gradual ethanol series, embedded in paraffine, cut into 3 to 4 µm thick sections using a microtome and subsequently stained with hematoxylin and eosin according to standard histological procedures. Digital images were taken (SLIDEVIEW VS200, EVIDENT Europe GmbH, Hamburg, Germany) and analyzed using the QuPath-0.5.0 software (https://qupath.github.io/) and the FIJI software with the BoneJ2 plugin (Domander et al. 2021). On the cross-section of the bone, the inner (h) and outer horizontal (H) as well as inner (b) and outer vertical diameters (B) were determined (QuPath). These values were used to calculate the wall thickness (wall thickness [mm] = [(B + H)-(b + h)]/4), the cortical wall area (cortical wall area [mm^2^] = π/4 × [(B × H) – (b × h)]) and the bone index (bone index = [(H − h)/H] + [(B − b)/B]) (Skiba et al. 2017; Vötterl et al. 2020). The number of osteoclasts was counted 10 times on a 1,000 µm long bone surface located at the transition from the bone cortex to the bone marrow (QuPath). Six regions of interest (ROI; 250 µm × 250 µm) were randomly placed within the bone cortex. In each ROI, the number of osteocytes was counted (QuPath) and the total bone area as well as the ratio of bone area to ROI were determined (FIJI with BoneJ2; Bouxsein et al. 2010; Moe et al. 2011; Oikonomidou et al. 2016). On the longitudinal section, the width of the growth plate and its reserve, proliferative and hypertrophic zone were measured 10 times (QuPath; Nilsson et al. 2005; Voller et al. 2020).

### Isolation of RNA and quantitative PCR

Total RNA was isolated from 25 mg of bone marrow using mechanical homogenization and the RNeasy Mini QIAcube Kit (Qiagen, Hilden, Germany) according to an adapted protocol (Vötterl et al. 2020). The RNA was treated with DNase I (Invitrogen TURBO DNA-free Kit; Thermo Fisher Scientific Inc., Waltham, MA, USA) to remove genomic DNA. The RNA quality and quantity were determined using the Invitrogen Qubit 4 Fluorometer using the Invitrogen Qubit RNA HS Assay Kit and the Invitrogen Qubit RNA IQ Assay Kit (Thermo Fisher Scientific Inc., Waltham, MA, USA). If the RNA integrity number was below 8, the RNA was re-isolated from the respective sample. For the synthesis of complementary DNA (cDNA), the High Capacity cDNA RT kit was used according to the manufacturer’s protocol (High-Capacity cDNA Reverse Transcription Kit; Thermo Fisher Scientific Inc., Waltham, MA, USA). Primers for amplification of target genes and reference genes were either verified or newly designed using PrimerBLAST (www.ncbi.nlm.nih.gov/tools/primer-blast/; Table S2). Twelve target genes (*FGF23*, fibroblast growth factor; *KLOTHO*, α-klotho; *OCN*, osteocalcin; *ALP*, alkaline phosphatase; *OPG*, osteoprotegerin; *RANKL*, receptor activator of NF-κB ligand; *VDR*, vitamin D receptor; *CaSR*, calcium-sensing receptor; *SLC20A1*, sodium-phosphate cotransporter 1; *SLC20A1*, sodium-phosphate cotransporter 2; *SLC34A2*, sodium-phosphate cotransporter 2a; *SLC34A2*, sodium-phosphate cotransporter 2b) and five reference genes (*ACTG*, γ-actin; *B2M*, β2-microglobulin; *GAPDH*, glyceraldehyde-3-phosphate dehydrogenase; *HPRT*, hypoxanthine-guanine phosphoribosyl transferase; *OAZ1*, ornithine decarboxylase antizyme 1) were amplified. The qPCR was performed using a pipetting robot (epMOTION 5075 TMX, Eppendorf SE, Hamburg, Germany). Amplification and quantification of cDNA was performed using the Innu MIX qPCR DS Green Standard (IST Innuscreen, Berlin, Germany) on the qTower384 qPCR system (Analytik Jena, Jena, Germany). Each standard and sample reaction of 10 μL contained 7 μL of master mix (innuMIX qPCR DS Green Standard), forward and reverse primers (200-400 nM each), and 3 μL of cDNA template (25 ng). After a first denaturation step at 95°C for 2 min, 40 cycles at 95°C for 30 s followed for primer annealing and elongation at 60°C for 30 s were conducted. The fluorescence was measured in all cycles and a melting curve analysis was performed to verify the specificity of the PCR product. To normalize raw gene expression and determine ΔCt values for relative gene expression levels, the geometric mean of the most stably expressed reference genes (*ACTG*, *B2M* and *GAPDH*) was used. Afterward, the 2^-ΔΔCt^ method was used to calculate relative gene expression. The sample with the highest expression indicated by the lowest ΔCt value of the respective target gene was subtracted from the ΔCt values resulting in the ΔΔCt values. All primers and their respective amplification efficiencies (E = 10^(-1/slope)^) are shown in Table S2.

### Statistical analysis

The data was tested for normal distribution using the Shapiro–Wilk test in SAS (version 9.4, SAS Inst. Inc., Cary NC, USA). Bone and serum data were subjected to ANOVA using the MIXED procedure in SAS. A random model was used to test the fixed effect of increasing age (ie DoL) of piglets on parameters. Sex was included in the model as covariate, but sex effects were rare. The random effect was animal, whereas DoL was the experimental unit. Degrees of freedom were approximated by the Kenward-Rogers method (ddfm = kr). The Bonferroni post hoc test was applied to perform multiple pairwise comparisons among least-square means. Data were reported as least-square means ± standard error of the mean (SEM). A significant difference was defined at *P *≤ 0.05 and trends at 0.05 < *P* ≤ 0.10. Descriptive statistics of body weight of piglets sampled on the consecutive sampling days was performed using PROC MEANS in SAS. Polynomial contrasts were performed to test linear, quadratic and cubic effects of age preweaning from DoL 3 to 28 (*P_prew_*) as well as linear and quadratic effects of age postweaning between DoL 28 and 35 (*P_postw_*). As bones produce BALP, OCN and FGF23 and carry the VDR, Pearson correlation coefficients of serum levels and mRNA expression in metacarpal bones of these parameters were calculated using PROC CORR in SAS separately for the suckling (DoL 3 to 28) and early postweaning phase (DoL 28 to 35). Only correlation coefficients |*r*| > 0.35 and *P *< 0.05 were considered physiologically relevant.

## Results

### Metacarpal bone development

The descriptive statistics for body weight and body weight gain of the piglets that were sampled on the consecutive days of life can be found in Table S3. The metacarpal bones linearly increased in size from DoL 3 to 28 (*P_prew_* < 0.001; Table 1). Consequently, bone metric parameters, such as bone weight, length, vertical and horizontal internal and external diameters cross sections and the corresponding cross-sectional area as well as dry matter content (*P_prew_* < 0.05) and, as trend, bone density (*P_prew_* = 0.054), linearly increased during the suckling phase. The cortical wall thickness and the cortical index, in turn, showed a cubic response between DoL 3 and 28 (*P_prew_* < 0.001), whereas crude ash quadratically changed with increasing age being highest on DoL 14 (*P_prew_* < 0.05). Bone histological parameters indicated quadratic effects of age on the total width of the growth plate together with its proliferative and reserve zones between DoL 3 and 28 with greater areas on DoL 7 (*P_prew_* < 0.01). Moreover, polynomial contrasts indicated a linear increase in the bone cortex, the number of osteocytes (based on ROI) as well as the number of osteoclasts from DoL 3 to 28 (*P_prew_* < 0.05).

**Table 1:** Age-related development of bone metrics and histological parameters in metacarpal bones of suckling and newly weaned piglets.^1^,^2^

Item	Day								Effect of day, *P*-value	Polynomial contrasts				
										Preweaning (d 3–28), *P_prew_*			Postweaning (d 28–35), *P_postw_*	
	3	7	14	21	28	31	35	SEM		Linear	Quadr.	Cubic	Linear	Quadr.
**Geometry parameters**														
** Weight, g**	1.4^d^	1.9 ^cd^	2.8^c^	3.9^b^	5.0^a^	4.6^ab^	5.1^a^	0.25	<0.001	<0.001	0.118	0.589	0.847	0.320
** Length, mm**	23.8^d^	26.1^d^	32.2^c^	35.0^bc^	37.9^ab^	37.8^ab^	38.9^a^	0.69	<0.001	<0.001	0.429	0.081	0.356	0.532
** b (mm) vertical internal diameter**	4.1^c^	5.1 ^cd^	5.7^bc^	6.4^b^	7.8^a^	7.2^ab^	7.5^a^	0.22	<0.001	<0.001	0.184	0.081	0.373	0.232
** B (mm); vertical external diameter**	6.0^d^	6.6^d^	7.9^c^	8.7^b^	10.0^a^	9.4^a^	9.6^a^	0.24	<0.001	<0.001	0.222	0.700	0.247	0.235
** h (mm); horizontal internal diameter**	4.7^d^	5.7^c^	6.0^bc^	6.7^b^	8.2^a^	7.7^a^	8.2^a^	0.21	<0.001	<0.001	0.079	0.015	0.897	0.203
** H (mm); horizontal external diameter**	6.8^d^	7.4^d^	8.6^c^	9.8^b^	11.2^a^	10.7^ab^	11.1^a^	0.22	<0.001	<0.001	0.048	0.395	0.854	0.211
** Cortical wall thickness, mm**	1.05^bc^	0.81^c^	1.19^ab^	1.33^a^	1.28^ab^	1.30^a^	1.26^ab^	0.054	<0.001	<0.001	0.663	<0.001	0.788	0.872
** Cross-sectional area, mm^2^**	17.5^c^	15.6^c^	26.4^b^	33.5^ab^	37.2^a^	34.8^a^	35.7^a^	1.69	<0.001	<0.001	0.216	0.003	0.548	0.449
** Cortical index**	0.63^a^	0.48^b^	0.58^a^	0.60^a^	0.48^b^	0.52^ab^	0.49^b^	0.021	<0.001	0.005	0.449	<0.001	0.970	0.379
** Density, g/cm^3^**	1.17	1.17	1.18	1.18	1.19	1.17	1.16	0.006	0.056	0.054	0.902	0.737	0.001	0.173
** Dry matter, %**	28.2^c^	29.9^c^	34.8^b^	37.2^b^	41.0^a^	40.3^a^	38.4^a^	0.73	<0.001	<0.001	0.477	0.370	0.053	0.606
** Crude ash, % DM**	36.8	36.4	40.9	39.8	38.1	38.7	38.4	1.07	0.067	0.058	0.030	0.079	0.859	0.786
**Histological parameters**														
** Total width of the growth plate, µm**	1,485^b^	1,714^a^	1,481^b^	1,188^c^	1,104^c^	903^d^	981^cd^	43.04	<0.001	<0.001	<0.001	<0.001	0.020	0.003
** Reserve zone of the growth plate, µm**	982^ab^	1,145^a^	926^b^	675^c^	604^c^	482^d^	511^cd^	37.52	<0.001	<0.001	0.001	<0.001	0.042	0.050
** Proliferative zone of the growth plate, µm**	341^b^	400^a^	387^a^	334^b^	319^b^	254^c^	271^c^	8.85	<0.001	0.001	<0.001	0.001	0.001	0.001
** Hypertrophic zone of the growth plate, µm**	162	168	168	179	182	168	198	9.88	0.157	0.130	0.890	0.952	0.209	0.050
** Total area of the cortex, mm^2^**	30.63^b^	41.02^ab^	37.16^ab^	42.74^ab^	42.93^a^	40.94^ab^	40.14^ab^	2.657	0.032	0.001	0.229	0.247	0.555	0.885
** Cortex area: ROI-ratio, mm^2^/ROI**	0.49	0.66	0.60	0.68	0.68	0.65	0.64	0.043	0.036	0.005	0.120	0.861	0.565	0.881
** Osteocytes, number/nm^2^ of cortex area**	0.80	0.60	0.80	0.90	1.10	0.90	1.10	0.16	0.404	0.006	0.159	0.343	0.950	0.407
** Osteocytes, number/nm^2^ of ROI**	0.36^d^	0.42 ^cd^	0.50^c^	0.62^b^	0.68^a^	0.55^b^	0.55^b^	0.02	<0.001	<0.001	0.715	0.196	<0.001	0.025
** Osteoclasts, number/nm**	1.1^d^	2.3^c^	3.6^b^	4.4^a^	4.7^a^	3.6^b^	4.3^ab^	0.19	<0.001	<0.001	0.005	0.242	0.154	<0.001

Quadr. = quadratic contrast.

1 Values are presented as least squares means ± SEM. Piglets were weaned on d 28 of life. On each day of life, 10 piglets (5 males and 5 females) were sampled.

2 The present value represents the average value for 6 ROI of 250 µm × 250 µm that were randomly placed within the bone cortex.

a,b,c,d Least squares means sharing no common superscript in the same row are significantly different (*P *< 0.05) according to Tukey’s method.

Postweaning, bone weight, length, vertical and horizontal internal and external diameters of cross sections, the cross-sectional area, cortical wall thickness and the cortical index remained the same size on d 31 and 35 compared to d 28 of life (*P_postw_* > 0.10). While the crude ash content remained similar, bone density and dry matter tended to linearly decrease from DoL 28 to 35 (*P_postw_* = 0.053). By contrast, the total width of the growth plate, and the proliferative and reserve zones quadratically changed postweaning being lowest on DoL 31 (*P_postw_* < 0.01). Although the hypertrophic zone of the growth plate did not change in size from DoL 3 to 28, there was a quadratic effect of age postweaning indicating a reduced size of the hypertrophic zone on DoL 31 (*P_postw_* = 0.05). A similar quadratic response after weaning was observed for the number of osteoclasts being lower on DoL 31 (*P_postw_* < 0.001), whereas the number of osteocytes (based on ROI) linearly decreased postweaning (*P_postw_* < 0.001).

Pearson correlations between serum parameters and their relative mRNA expression in metacarpal bones only showed a positive correlation between serum OCN and its mRNA expression in the suckling phase (*r *= 0.53; *P *< 0.001) but not postweaning.

### Serum parameters

Age influenced all measured parameters in serum related to mineral homeostasis (Table 2). A cubic and a quadratic effect of age on serum Ca and phosphate were observed from DoL 3 to 28, respectively (*P_prew_* < 0.001), resulting in a quadratic change in the serum Ca: phosphate ratio in the suckling phase with the lowest Ca: phosphate ratio on DoL 14 (*P_prew_* < 0.001). Serum VitD and PTH linearly increased during the suckling phase (*P_prew_* < 0.001). Serum ALP, BALP and FGF23, in turn, linearly decreased during the suckling period (*P_prew_* < 0.001). Serum OCN showed a quadratic effect during the suckling phase with its highest value on DoL 14 (*P_prew_* < 0.001). Postweaning, serum Ca and P quadratically changed from DoL 28 to 35 with lower values on DoL 31 (*P_postw_* < 0.001). Serum VitD levels continued to linearly increase, whereas those of PTH, ALP and BALP linearly decreased from DoL 28 to 35 (*P_postw_* < 0.05). Serum OCN and FGF23 remained stable from DoL 28 to 35.

**Table 2:** Age-related development of serum parameters for bone metabolism in suckling and newly weaned piglets.^1^

Item	Day									Polynomial contrasts, *P*-value				
									Effect of day, *P*-value	Preweaning (d 3–28), *P_prew_*			Postweaning (d 28–35), *P_postw_*	
	3	7	14	21	28	31	35	SEM		Linear	Quadr.	Cubic	Linear	Quadr.
**Calcium, mmol/L**	2.63^bc^	2.90^a^	2.75^ab^	2.63^bc^	2.69^b^	2.48^c^	2.56^bc^	0.042	<0.001	0.284	0.027	<0.001	0.019	0.002
**Phosphor, mmol/L**	2.18^b^	2.84^a^	3.22^a^	2.69^ab^	2.77^ab^	2.37^b^	2.44^ab^	0.093	<0.001	0.002	<0.001	0.007	0.006	0.019
**Ca/P ratio**	1.25^a^	1.02^ab^	0.84^b^	0.98^ab^	0.99^ab^	1.06^a^	1.07^a^	0.041	<0.001	<0.001	<0.001	0.210	0.091	0.373
**PTH, pg/mL**	12.0^b^	9.43^b^	20.8^ab^	29.3^ab^	41.1^a^	37.7^a^	16.1^b^	5.24	<0.001	<0.001	0.218	0.541	0.017	0.290
**VitD, ng/mL^2^**	5.46^c^	5.74^c^	6.70^bc^	7.59^ab^	7.13^bc^	7.85^ab^	9.13^a^	0.378	<0.001	<0.001	0.206	0.054	0.005	0.617
**FGF23, pg/mL**	620^a^	204^b^	117^b^	177^b^	97^b^	106^b^	89^b^	78.50	<0.001	<0.001	0.013	0.080	0.855	0.761
**Alkaline phosphatase, U/L**	1,868^a^	1,839^a^	960^ab^	466^b^	343^b^	284^b^	233^b^	211.4	<0.001	<0.001	0.834	0.130	<0.001	0.842
**BALP, µg/L**	375^a^	408^a^	238^b^	142^bc^	88^c^	79^c^	61^c^	29.12	<0.001	<0.001	0.443	0.029	<0.001	0.359
**OCN, ng/mL**	30.0^b^	47.3^a^	56.1^a^	48.4^a^	48.0^a^	47.4^a^	51.8^a^	2.55	<0.001	<0.001	<0.001	0.079	0.198	0.335

1 Values are presented as least squares means ± SEM. Piglets were weaned on d 28 of life. On each day of life, 10 piglets (5 males and 5 females) were sampled.

2 The 25-hydroxy vitamin D_3_ and D_2_ were determined.

Quadr. = quadratic contrast.

a,b,c Least squares means sharing no common superscript in the same row are significantly different (*P *< 0.05) according to Tukey’s method.

### Messenger RNA expression in bone marrow

The relative mRNA expression of *VDR, OCN, OPG*, and *RANKL* linearly increased from DoL 3 to 28 (*P_prew_* < 0.01; Table 3). By contrast, the mRNA expression of *FGF23* and *KLOTHO* linearly decreased from DoL 3 to 28 with the strongest decrease in the mRNA expression level from DoL 7 to 14 (*P_prew_* < 0.001). Postweaning, the mRNA expression of *OCN* linearly increased, whereas that of *OPG* and *KLOTHO* responded quadratically to age from DoL28 to 35 being lower and higher on DoL 31, respectively (*P_postw_* < 0.05). The mRNA expression of *VDR, FGF23*, and *RANKL*, in turn, remained stable postweaning. The mRNA expression of the sodium-phosphate co-transporters showed different age-related patterns. Mainly, the mRNA expression of *SLC20A1* linearly decreased (*P_prew_* ≤ 0.05), whereas that of *SLC20A2* linearly increased from DoL3 to 28 and continued to linearly increase from DoL 28 to 35 (*P_postw_* = 0.009). Only the mRNA expression of *OCN* and its serum levels positively correlated for the suckling phase (*r *= 0.53; *P *< 0.001) but not postweaning.

**Table 3:** Age-related development of relative mRNA expression of genes for mineral metabolism in marrow of metacarpal bones in suckling and newly weaned piglets.^1^

Gene of interest		Day								Effect of day, *P*-value	Polynomial contrasts				
											Preweaning (d 3–28), *P_prew_*			Postweaning (d 28–35), *P_postw_*	
		3	7	14	21	28	31	35	SEM		Linear	Quadr.	Cubic	Linear	Quadr.
***VDR***	Vitamin D receptor	0.23^c^	0.24^c^	0.29^bc^	0.32^bc^	0.47^a^	0.46^ab^	0.51^a^	0.040	<0.001	<0.001	0.028	0.382	0.651	0.640
***FGF23***	Fibroblast growth factor-23	0.30^ab^	0.36^a^	0.11^b^	0.03^b^	0.02^b^	0.02^b^	0.04^b^	0.046	<0.001	<0.001	0.827	0.034	0.211	0.500
***KLOTHO***	α-Klotho	0.45^ab^	0.61^a^	0.57^a^	0.31^bc^	0.18^c^	0.27^c^	0.18^c^	0.059	<0.001	<0.001	0.003	0.119	0.971	0.044
***OCN***	Osteocalcin	0.08^b^	0.16^b^	0.28^b^	0.24^b^	0.44^a^	0.53^a^	0.62^a^	0.048	<0.001	<0.001	0.595	0.130	0.042	0.993
***OPG***	Osteoprotegerin	0.28^b^	0.31^ab^	0.46^ab^	0.58^ab^	0.46^ab^	0.34^ab^	0.60^a^	0.070	0.008	0.005	0.211	0.114	0.217	0.043
***RANKL***	Receptor activator of nuclear factor kappa-Β ligand	0.27^bc^	0.22^c^	0.35^b^	0.40^b^	0.40^b^	0.44^ab^	0.50^a^	0.044	<0.001	<0.001	0.894	0.046	0.184	0.860
***CASR***	Ca-sensing receptor	0.13	0.24	0.15	0.15	0.20	0.21	0.29	0.067	0.486	0.750	0.854	0.110	0.551	0.719
***ALP***	Bone-specific alkaline phosphatase	0.31^ab^	0.16^b^	0.31^ab^	0.31^ab^	0.38^ab^	0.36^ab^	0.44^a^	0.055	0.031	0.052	0.098	0.146	0.528	0.488
***SLC20A1***	Sodium-phosphate cotransporter 1	0.38^b^	0.60^a^	0.39^b^	0.19^c^	0.14^c^	0.14^c^	0.12^c^	0.031	<0.001	<0.001	<0.001	<0.001	0.440	0.772
***SLC20A2***	Sodium-phosphate cotransporter 2	0.38^b^	0.37^b^	0.47^ab^	0.52^ab^	0.43^ab^	0.50^ab^	0.67^a^	0.049	0.001	0.050	0.150	0.055	0.009	0.528
***SLC34A1***	Sodium-phosphate cotransporter 2a	0.32	0.33	0.30	0.23	0.46	0.34	0.40	0.059	0.128	0.372	0.078	0.088	0.583	0.355
***SLC34A2***	Sodium-phosphate cotransporter 2b	0.21	0.35	0.19	0.21	0.18	0.24	0.23	0.047	0.252	0.179	0.406	0.099	0.446	0.511

Quadr. = quadratic contrast.

1 Values are presented as least squares means ± SEM. Piglets were weaned on d 28 of life. On each day of life, 10 piglets (5 males and 5 females) were sampled.

a,b,c Least squares means sharing no common superscript in the same row are significantly different (*P *< 0.05) according to Tukey’s method.

## Discussion

The present study provides novel data for serum parameters related to bone formation, bone geometry and histomorphology as well as mRNA expression in metacarpal bones of suckling and newly weaned pigs. The studied parameters in serum and bone showed clear age-dependent patterns and the effect of weaning, which may be useful as reference values for clinical diagnostics and future studies especially in suckling piglets. Although bones produce factors relevant for growth and renewal and express the VDR (Robling and Bonewald 2020), associations between mRNA expression and serum value only existed for OCN. Several reasons may explain the lack of associations such as the type of bone, half-life of the factors in the blood and translational changes from mRNA to functional protein. We investigated the age-related development of metacarpal bones as they have been often used for diagnostic purposes in the past and for studying dietary effects in pigs (Hagemoser et al. 2000; Sobol et al. 2024). They are relatively small bones and thus easier to remove from the surrounding tissue than larger bones, which is helpful in clinical diagnostics. However, their mRNA expression and synthesis of hormonal factors may differ from those in long bones, such as the femur, as indicated by recent observation in nursery and growing pigs (Williams et al. 2023, 2024). The mRNA expression in the bone marrow may also diverge from other areas of the bone (eg growth plate), which may be worth to follow up in future studies.

Suckling piglets have a high growth intensity, doubling their birth weight in the first 14 d of life (Yosi et al. 2024). Accordingly, most of the bone geometry parameters as well as the numbers of osteocytes (based on ROI) and osteoclasts as indicators for bone maturation showed a linear increase in size during the suckling phase. In fact, the number of osteocytes may be used as proxy for the stage of mineralization of the bone as osteocytes are cells embedded within the hard mineralized matrix and are a sign of mature bone matrix (Robling and Bonewald 2020). Osteocytes are also an important source of factors orchestrating growth and renewal of bone tissue (Robling and Bonewald 2020). The quadratic effect of age on several histological parameters including the total width of the growth plate as well as of its proliferative and reserve zones supported a higher growth intensity in the earlier suckling phase. The proliferative zone of the growth plate is responsible for longitudinal bone growth (Haraguchi et al. 2019), whereas the reserve zone provides the cellular supply for the proliferative and hypertrophic zones. The consistent size of the hypertrophic zone throughout the suckling phase suggested a uniform and continuous longitudinal growth of the bone.

Bone ALP produced by osteoblasts as part of the total ALP activity is an important enzyme for bone formation by providing phosphates for extracellular bone mineralization (Halling Linder et al. 2017). Therefore, higher serum BALP and ALP levels in the first week of life compared to the later suckling phase may have stimulated the bone mineralization processes after birth. Of note, the mRNA expression of BALP in the metacarpal bones showed a different age-related profile compared to the serum level, which may be due to different gene transcription between types of bones, or due to reduced translation of the mRNA into active protein. Another important factor for bone growth is the Ca-binding protein OCN, which is produced by osteoblasts. It plays an important role in the alignment of apatite crystallites parallel to collagen fibers (Manolagas 2020). Although the age-related response of the mRNA expression and serum OCN diverged, the observed positive correlation may be indicative that the metacarpal bones produced OCN. The quadratic effect of age on serum OCN supported the histological data, suggesting a stronger stimulatory effect on bone maturation on DoL 14.

The Ca level in the blood is kept in a narrow range (Goldstein 1990). Accordingly, the serum Ca level was maintained between 2.63 and 2.90 mmol/L in the suckling phase. The hormones involved in the regulatory feedback loop showed a different, namely a linear, effect of age compared to serum Ca. Specifically, serum PTH, which secretion is triggered by a drop in blood Ca, and serum VitD, which is induced by PTH (Rillaerts et al. 2025), showed a linear increase over time during the suckling phase. A similar increase in plasma VitD has been previously reported in suckling pigs (Xu et al. 2025). It can be speculated whether the observed fluctuation in serum Ca were the result of the combined action of PTH and VitD.

The importance of VitD for bone growth was also indicated by the increasing mRNA expression of *VDR* in the metacarpal bone between DoL 3 and 28. Aside from triggering the renal absorption of Ca, PTH also triggers osteoblasts to produce RANKL, which activates the maturation of osteoclasts and thus is linked to bone resorption (Clarke 2008; Martin and Sims 2015). Accordingly, the mRNA expression of *RANKL* and the number of osteoclasts linearly increased during the suckling phase. Similarly, the mRNA expression of *OPG* increased during the suckling phase, which may have limited bone resorption as a result of the higher numbers of osteoclasts with increasing age. Osteoprotegerin is produced by osteoblasts and acts as decoy receptor, by which it prevents the action of RANKL on osteoclastic precursor cells (Martin and Sims 2015).

Fibroblast-growth-factor 23 is an important regulatory factor in the phosphorus homeostasis. Its physiological role is to inhibit renal phosphate reabsorption, lowering the available phosphate levels for bone formation (Courbebaisse and Lanske 2018). In line with this role, linearly decreasing serum FGF23 levels and mRNA expression levels in metacarpal bones during the suckling phase supported retention of phosphate, becoming available for bone growth. In contrast to FGF23, serum phosphate levels showed a quadratic effect of age, probably reflecting the utilization of phosphate for bone growth and regulation of excretion. The signal transduction of FGF23 on phosphate reabsorption in the kidneys requires the transmembrane protein α-Klotho (Quarles 2019). Its mRNA expression in the metacarpal bones showed a slightly shifted temporal pattern as that of *FGF23*, which may have contributed to the quadratic age-related changes in serum phosphate during the suckling phase. Mechanistically, this feedback may be mediated via sodium-dependent phosphate transporters, especially sodium-phosphate cotransporter 2, as shown in a mouse model (Bon et al. 2018). In contrast to the mouse model, the feedback may have been transmitted via mRNA expression of *SLC20A1* in the present study as its mRNA corresponded better to the decreasing P levels in the blood during the suckling phase than the mRNA expression of *SLC20A2* in the present study. Moreover, the transcription of PTH, VitD and FGF23 are linked via positive and negative feedback loops (Rillaerts et al. 2025). The active 1,25-bihydroxy VitD induces *FGF23* transcription in osteoblasts and osteocytes (Rillaerts et al. 2025), whereas FGF23 inhibits the conversion of the 25-hydroxy-VitD into 1,25-dihydroxy-VitD (Shimada et al. 2004; Courbebaisse and Lanske 2018). The extent to which these feedback loops contributed to the opposite temporal patterns between VitD and FGF23 is difficult to infer from the available data. We only determined 25-hydroxy-VitD, which is the storage form of VitD in the liver and commonly used as proxy for VitD status, but not the biologically active 1,25-dihydroxy-VitD. Sow milk is rich in 25-hydroxy VitD (Xu et al. 2025). Consequently, increasing intake of sow milk by the piglets with increasing age probably directly contributed to the measured serum VitD in the present study, whereas the cholecalciferol in the milk replacer and prestarter diet needed first to be hydroxylated in the liver (Rillaerts et al. 2025).

Postweaning, the lack of increase in bone size and the decrease in bone density corresponded to the observed growth stagnation in these piglets probably due to reduced feed consumption after weaning (Yosi et al. 2024). Increased cortisol levels due to the experienced stress after weaning may have further diverted energy and nutrients from growth (Wensley et al. 2023). Results for histological parameters, such as the width of the growth plate, its proliferative, reserve and hypertrophic zones, osteocytes (based on ROI) and osteoblasts, from DoL 28 to 35 supported reduced longitudinal bone growth and interrupted bone maturation in the first days after weaning. However, the quadratic effect of age on these parameters also suggested the resumption of bone development 1 wk after weaning. At mRNA expression level, quadratic effects of age on the expression of *KLOTHO* and *OPG* may suggest lower utilization of phosphate for bone growth and lower activity of osteoblasts, respectively. The mRNA expression of *OCN*, in turn, appeared not to be affected by weaning, indicating a continued increase in bone strength at least in the metacarpal bone. However, results for OCN in serum supported the findings for bone geometry parameters of stagnated growth. In line with that, the quadratic response to age postweaning of serum Ca and phosphate with lower levels on DoL 31 may have reflected the reduced feed intake directly after weaning. By increasing the renal reabsorption and intestinal absorption of Ca (Rillaerts et al. 2025), the increasing level of serum VitD postweaning may have resulted in the restoration of the serum Ca level on DoL35. Interestingly, serum levels of PTH and VitD showed an opposite relation to age. The linear decrease in serum PTH from DoL 28 to 35 (especially from DoL 31 to 35) suggested sufficient Ca availability. The stronger decrease in serum PTH from DoL 31 to 35 may have been related to increased dietary Ca intake and the action of VitD to increase intestinal absorption.

In conclusion, the present study provides valid data for temporal patterns of serum factors related to bone growth and their association with bone metrics, histology and mRNA expression in suckling and newly weaned piglets. The regulatory factors partly showed diverging temporal dynamics in serum and mRNA expression in the bone, which may be indicative for fine-tuning of bone mineralization and regulatory feedback loops. However, they may be also explained by differences in the contribution of hormonal factors to blood levels depending on the bone, which should be investigated in future studies. While weaning caused bone growth stagnation, data at serum and bone level indicated resumption of bone mineralization 1 wk after weaning. Overall, the present data are useful for future diagnostic investigations in suckling piglets to refine reference values under different nutritional interventions and health conditions.

## Supplementary Material

skag331_Supplementary_Data

## Abbreviations

ACTG: γ-actin
ALP: alkaline phosphatase
B2M: β2-microglobulin
BALP: bone-specific alkaline phosphatase
Ca: calcium
CaSR: calcium-sensing receptor
cDNA: complementary DNA
DoL: day of life
ELISA: enzyme-linked immunosorbent assay
FGF23: fibroblast growth factor 23
GAPDH: glyceraldehyde-3-phosphate dehydrogenase
HPRT: hypoxanthine-guanine phosphoribosyl transferase
OAZ1: ornithine decarboxylase antizyme 1
OCN: osteocalcin
OPG: osteoprotegerin
P: phosphorus
PTH: parathormone
RANKL: receptor activator of NF-κB ligand
ROI: region of interest
SLC20A1: sodium-phophate cotransporter 1
SLC20A2: sodium-phosphate cotransporter 2
SLC34A1: sodium-phosphate cotransporter 2a
SLC34A2: sodium-phosphate cotransporter 2b
VDR: vitamin D receptor
VitD: vitamin D
