# Supplementary material for: Postnatal development of bones and related hormonal factors in piglets
Source: J Anim Sci. 2026 Oct 10;104:skag331. doi: 10.1093/jas/skag331 (PMC13700059; doi:10.1093/jas/skag331)
Supplement: Supplementary file 1 [file skag331_supplementary_data.docx]

**Table S1**. Analyzed nutrient composition of the milk replacer and prestarter diet for piglets (adapted from Yosi et al., 2024)

| Chemical composition, % dry matter | Milk replacer^1^ | Prestarter diet^2,3^ |
| --- | --- | --- |
| Dry matter, % | 91.6 | 89.8 |
| Crude protein | 23.3 | 18.2 |
| Crude fiber | 1.9 | 4.9 |
| Ether extract | 10.3 | 7.3 |
| Crude ash | 6.8 | 5.1 |
| Nitrogen-free extract | 57.9 | 65.0 |
| Starch | 25.4 | 42.5 |
| Metabolizable energy, MJ/kg | 16.9 | 15.4 |
| Macro minerals, % |  |  |
| Calcium | 0.80 | 0.73 |
| Phosphorus | 0.80 | 0.72 |
| Magnesium | 0.20 | 0.21 |
| Potassium | 1.09 | 0.67 |
| Sodium | 0.50 | 0.24 |
| Trace minerals, ppm |  |  |
| Iron | 264 | 333 |
| Manganese | 87 | 118 |
| Zinc | 129 | 182 |
| Copper | 146 | 152 |

^1^NuriStart Sweet, BIOMIN Holding GmbH, Part of dsm-firmenich, Getzersdorf, Austria. Ingredients: wheat flour, whey protein concentrate, whey powder, extruded soybeans, puffed corn, rolled oats, soy protein concentrate, sugar, dextrose, palm oil, monocalcium phosphate,

coconut oil, sodium chloride, calcium carbonate, and salmon oil. Technological additives per kg: 400 mg sepiolite (E562), 3,000 mg citric acid (E330), 0.07 mg propyl gallate (E310), and 1 mg butyrylhydroxy toluol (E321). Zootechnical additives per kg: 200 FXU endo-1,4-beta-xylanase EC 3.2.1.8; 1,000 FYT 6-phytase EC 3.1.3.26. Nutritional additives per kg: 16,000 IE of vitamin A, 2,000 IE of vitamin D3, 150 mg of vitamin E, 200 mg of vitamin C, 195 mg of Fe as iron (II) sulfate monohydrate, 2.0 mg of I as calcium iodate anhydrous, 0.40 mg of Se as sodium selenite, 60 mg of Mn as manganese (II) oxide, 140 mg of Cu as copper (II) sulfate pentahydrate, and 100 mg Zn as zinc oxide.

^2^Königshofer GmbH, Ebergassing, Austria. Ingredients: oat flakes, barley, wheat, whey powder, soy protein concentrate, wheat bran, sucrose, soybean meal, soy oil, corn gluten, L-cellulose, monocalcium phosphate, fish oil, sodium chloride, magnesium phosphate, calcium carbonate and molasses. Technological additives per kg: 400 mg sepiolite (E562), 3,000 mg citric acid (E330), 0.07 mg propyl gallate (E310), and 1 mg butyrylhydroxy toluol (E321). Zootechnical additives per kg: 1,000 IU of phytase, 1,500 EPU of xylanase, 11 mg of beta hydroxy acid, 21 mg of butylated hydroxytoluene, and 11 mg of propyl gallate. Nutritional additives per kg: 16,000 IE of vitamin A, 2,000 IE of vitamin D3, 200 mg of vitamin E, 120 mg of Fe as iron (II) sulfate, 140 mg of Cu as copper (II) sulfate, 100 mg of Zn as zinc oxide, 60 mg of Mn as manganes (II) oxide, 1.5 mg of I as calcium iodate, 0.5 mg of Se as sodium selenite.

^3^Piglets were offered the milk replacer from day of life (DoL) 3 to 23; and combinations of milk replacer and prestarter diet with a ratio of 70:30 (w/w) on DoL24, 50:50 (w/w) on DoL25, and 30:70 (w/w) on DoL26, respectively; and 100% prestarter diet on DoL27 and 28.

**Table S2.** Primer for quantitative PCR in metacarpal bone marrow

| Genes | Accession number | Primer sequence (5’ to 3’) | | Efficien-cies (%) | Amplicon size (bp) | Reference |
| --- | --- | --- | --- | --- | --- | --- |
| *Reference genes^1^* | | | | | | |
| *ACTG* | XM_003357928.4 | F: | GGGCATCCTGACCCTCAAG | 100 | 89 | (Metzler-Zebeli et al., 2015) |
|  |  | R: | TGTAGAAGGTGTGATGCCAGATCT |  |  |  |
| *B2M* | NM_213978.1 | F: | CCCCCGAAGGTTCAGGTT | 101 | 66 | (Metzler-Zebeli et al., 2015) |
|  |  | R: | GCAGTTCAGGTAATTTGGCTTTC |  |  |  |
| *GAPDH* | NM_001206359.1 | F: | GGCGTGAACCATGAGAAGTATG | 99 | 60 | (Metzler-Zebeli et al., 2015) |
|  |  | R: | GGTGCAGGAGGCATTGCT |  |  |  |
| *HPRT* | NM_001032376.2 | F: | AGAAAAGTAAGCAGTCAGTTTCATATCAGT | 98 | 131 | (Metzler-Zebeli et al., 2015) |
|  |  | R: | ATCTGAACAAGAGAGAAAATACAGTCAATAG |  |  |  |
| *OAZ1* | NM_001122994.2 | F: | TCGGCTGAATGTAACAGAGGAA | 99 | 70 | (Metzler-Zebeli et al., 2015) |
|  |  | R: | GAGCCTGGATTGGACGTTTAAA |  |  |  |
| *Target genes^2^* | | | | | | |
| *FGF23* | XM_001926525.4 | F: | CGCAGGCTTCGTGGTCATAA | 99 | 146 | (Vötterl et al., 2020) |
|  |  | R: | GGTACACGTCGTAGCCGTTC |  |  |  |
| *KLOTHO* | XM_021065566.1 | F: | GCTACAGCATCAGACGTGGA | 100 | 147 | (Kim et al., 2020) |
|  |  | R: | TCCCTTCTAGGGGCTGATTT |  |  |  |
| *OCN* | AY150038.1  XM_013996978.2  NM_001164004.1 | F: | ACGAGCTGGCTGATCACATC | 98 | 71 | (Vötterl et al., 2020) |
|  |  | R: | CTGCGAGGTCTAGGCTATGC |  |  |  |
| *ALP* | AH012163.2  MF955858.1  XM_021097682.1 | F: | CCCTTCACTGCCATCCTGTAC | 101 | 101 | (Chen et al., 2009) |
|  |  | R: | TGGTAGTTGTCGTGCGCATAG |  |  |  |
| *OPG* | XM_003481346.4 | F: | GACCTCTGTGAAAACAGTGTGC | 100 | 276 | (Vötterl et al., 2020) |
|  |  | R: | CTTCAGACTCTGTGTGACGGT |  |  |  |
| *RANKL* | AY606802.1  XM_001925694.6 | F: | CCAAGACGCCACGTACTTT | 101 | 71 | (Vötterl et al., 2020) |
|  |  | R: | AAACAACTCCACAAAATGGGGC |  |  |  |
| *VDR* | NM_001097414.1 | F: | TGGTTGGAAGTGTCTGGGAG | 98 | 117 | (Vötterl et al., 2020) |
|  |  | R: | GGGGTCAGGTAAGGAAGTGC |  |  |  |
| *CaSR* | NM_001278748.1 | F: | CATCAAGTTCCGAAACACGCCCAT | 98 | 172 | (Mahajan et al., 2011) |
|  |  | R: | GCAGAGCACGAAGCTAATGCCAAA |  |  |  |
| *SLC20A1* | XM_021087023.1 | F: | GCTTCTTTTGCTCGGTTGCTGAG | 98 | 229 | Newly designed |
|  |  | R: | CGGAGCAGAATTCCATGGCG |  |  |  |
| *SLC20A2* | XM_021078067.1 | F: | TCACTGTATCGTGGGGTCCA | 98 | 151 | Newly designed |
|  |  | R: | GATCAGTACAAACAGCACGCC |  |  |  |
| *SLC34A1* | XM_021082339.1 | F: | GGTGGCCAAGGTCATTCAGA | 99 | 120 | Newly designed |
|  |  | R: | GAACTGCTCTGGACGACGAA |  |  |  |
| *SLC34A2* | NM_001256772.1 | F: | GCTGGTTCGCCATCGTCT | 102 | 156 | Newly designed |
|  |  | R: | GGGACTGCAGGAGCTTAATG |  |  |  |

^1^*ACTG*, γ-actin; *B2M*, β2-microglobulin; *GAPDH*, glyceraldehyde-3-phosphate-dehydrogenase; *HPRT*, hypoxanthin-guanine phosphoribosyl transferase; *OAZ1*, ornithine decarboxylase antizyme 1.

*^2^FGF23*, fibroblast growth factor 23; *KLOTHO*, α-klotho; *OCN*, osteocalcin; *ALP*, alkaline phosphatase; *OPG*, osteoprotegerin; *RANKL*, receptor activator of NF-κB Ligand; *VDR*, vitamin D receptor; *CaSR*, calcium-sensing receptor; *SLC20A1*, Na^+^-Pi cotransporter 1; *SLC20A1*, Na^+^-Pi cotransporter 2; *SLC34A1*, Na^+^-Pi cotransporter 2a; *SLC34A2*, Na^+^-Pi cotransporter 2b.

**Table S3** Descriptive statistics (mean ± SD) for birth weight, body weight and body weight gain (BWG) of suckling and newly weaned piglets at sampling

| Item | N | Mean | SD |
| --- | --- | --- | --- |
| Day 3 of life |  |  |  |
| Birth weight (kg) | 10 | 1.4 | 0.12 |
| Weight day 3 (kg) | 10 | 1.6 | 0.18 |
| BWG day 1 to 3 | 10 | 0.2 | 0.09 |
| Day 7 of life |  |  |  |
| Birth weight | 10 | 1.5 | 0.14 |
| Weight day 7 | 10 | 2.5 | 0.43 |
| BWG day 1 to 7 | 10 | 1.0 | 0.37 |
| Day 14 of life |  |  |  |
| Birth weight | 10 | 1.5 | 0.30 |
| Weight day 14 | 10 | 4.4 | 1.13 |
| BWG day 1 to 14 | 10 | 2.9 | 0.91 |
| Day 21 of life |  |  |  |
| Birth weight | 10 | 1.5 | 0.22 |
| Weight day 21 | 10 | 6.2 | 1.83 |
| BWG day 1 to 21 | 10 | 4.7 | 1.70 |
| Day 28 of life |  |  |  |
| Birth weight | 10 | 1.5 | 0.23 |
| Weight day 28 | 10 | 8.5 | 1.66 |
| BWG day 1 to 28 | 10 | 7.0 | 1.46 |
| Day 31 of life |  |  |  |
| Birth weight | 10 | 1.5 | 0.29 |
| Weight day 31 | 10 | 7.9 | 1.23 |
| BWG day 1 to 31 | 10 | 6.4 | 1.28 |
| Day 35 of life |  |  |  |
| Birth weight | 10 | 1.5 | 0.33 |
| Weight day 35 | 10 | 8.4 | 1.53 |
| BWG day 1 to 35 | 10 | 6.9 | 1.30 |
